# Ten simple rules for working with other people’s code

**DOI:** 10.1371/journal.pcbi.1011031

**Published:** 2023-04-20

**Authors:** Charlie Pilgrim, Paul Kent, Kasra Hosseini, Ed Chalstrey

**Affiliations:** 1 The Mathematics of Systems CDT, Mathematics Department, The University of Warwick, Coventry, United Kingdom; 2 Experimental Psychology, University College London, London, United Kingdom; 3 Research Engineering Group, The Alan Turing Institute, London, United Kingdom; Carnegie Mellon University, UNITED STATES

This is a *PLOS Computational Biology* Methods paper.

## Introduction

Every time that you use a computer, you are using someone else’s code, whether that be an operating system, a word processor, a web application, research tools, or simply code snippets. Almost all code has some bugs and errors. In day to day life, these bugs are usually not too important or at least obvious when they do happen (think of an operating system crashing). However, in research, there is a perfect storm that makes working with other people’s code particularly challenging—research needs to be correct and accurate, researchers often use niche and non-commercial tools that are not built with best software practices, bugs can be subtle and hard to detect, and researchers have time pressures to get things done quickly. It is no surprise then that working with other people’s code is a common frustration for researchers and is even considered a rite of passage [1].

There are a wealth of resources addressing how to write better code in academia, including *PLOS ONE* “Ten Simple Rules” articles on reproducibility [2], documentation [3], and writing open-source software [4,5]. And there are calls for improving research reproducibility by publishing code [6] and ensuring it is reusable [7]. However, the inverse problem—working with other people’s code in research—does not receive as much attention. In industry, there are some resources for working with legacy code [8] (“legacy code” essentially translates to “other people’s code”). While industry software development practices are useful, they often cannot be applied blindly to research software development and instead need to be adapted [9]. Therefore, our approach is to bring together and integrate lessons from industry, existing literature, and the research experiences of the authors and their colleagues.

The rules in this article will be useful for academic researchers at all levels, from students to professors. Our focus is on pragmatic research efficiency, as opposed to absolute best practices that may introduce unrealistic time burdens. The rules are informed but opinionated, and as such, we encourage readers to use the rules as a starting point to think for themselves and do what works for them. Overall, if the reader can take one useful idea or thought from this article, then we will consider it successful.

### Rule 1: Clarify your goals

Before jumping in, it is a good idea to think about what you actually want to achieve. It might be that you just need to compute something or you might need to add some functionality that doesn’t currently exist in the code. Different aims require different approaches. For example, if you need to compute something, then the key requirement is to be confident that the computation is correct, while for adding functionality you will probably need to understand the code a bit more. Clarifying your goals can prevent you from doing more than you need to. At one extreme, you can almost always achieve your goals by going through the entire codebase and understanding every line of code, but this will usually be overkill and inefficient.

**Fig 1:**
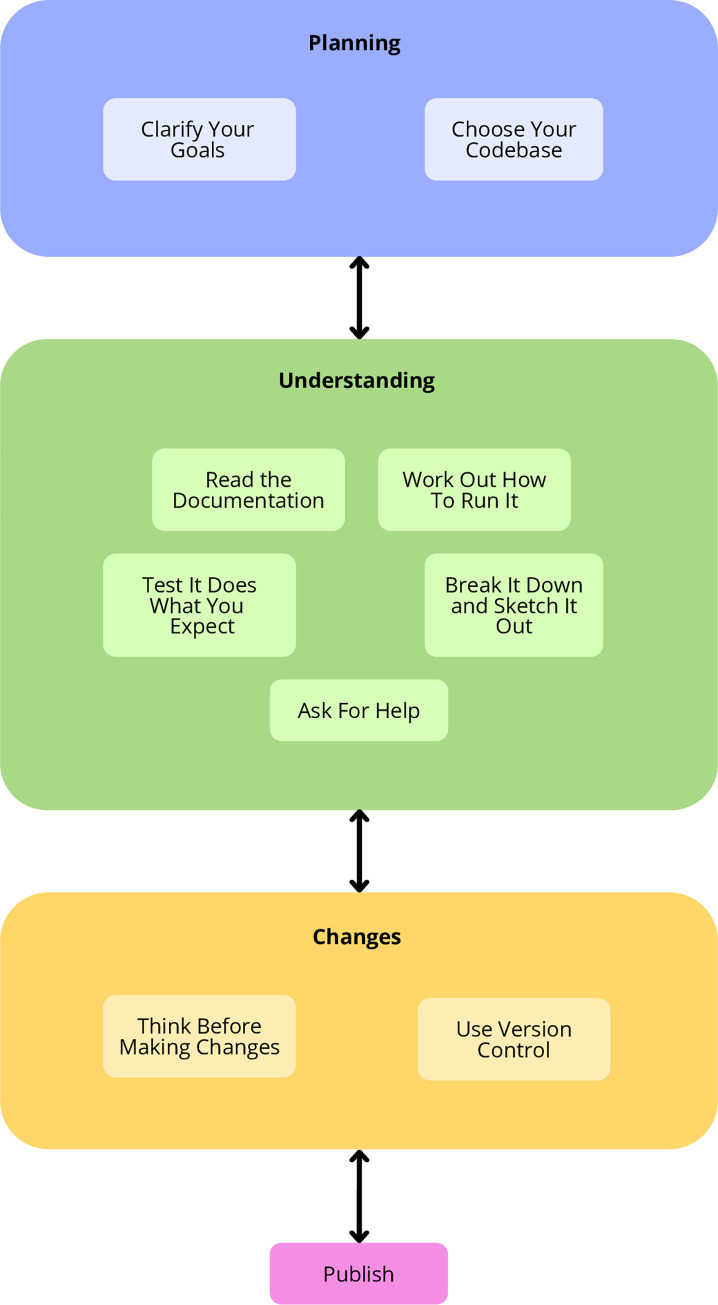
The 10 rules can be broken down into the categories of planning, understanding, changes, and publishing. Working with other people’s code is not a step by step process. As you build your understanding of the code, your goals may change and that will influence how, and whether, you make changes.

Clarifying your goals is a useful practice to do before jumping into a new codebase. Additionally, it is also useful to keep coming back to this question as you get to grips with the code (Fig 1). You may find that your original goal will take too much time, or more optimistically (if not realistically) perhaps you will find some shortcut that allows you to make progress with less work than you expected.

### Rule 2: Choose your codebase

Sometimes you will have little choice in which code you will have to work with, for example, if you have inherited an existing project. But often you will have a choice of which codebase to use to achieve your goals. It is therefore worthwhile searching on the internet for different options. To judge which codebase to use, you can look at the quality of the Documentation (see Rule 3: Read the documentation), how well maintained it is (e.g., the commit history on GitHub, see Rule 9: Use version control), and how active the community is (see Rule 7: Ask for help). You could also consider how accessible the codebase seems to be—can you make sense of the code? Relatedly, you might be able to choose between projects in different programming languages of which you have varying levels of familiarity.

A common dilemma is whether to rewrite the code from scratch or not. An astrophysicist colleague of the authors tells the story of FORTRAN code written in the 1970s that controls the positioning of telescopes and the problem of adding a new feature to this code. The question arises as to whether to (a) rewrite the entire thing in Python; or to (b) learn FORTRAN, get to grips with the codebase, and then, add the feature. This kind of choice involves many considerations that may only indirectly relate to the problem at hand, including whether it will be more desirable to (a) have the telescope control in Python for future changes; or to (b) know FORTRAN well enough to be able to work with other legacy systems. And while modern programming languages may make previously complicated tasks easier, it is usually unclear at the outset just how much work will be involved with starting again.

A key benefit of starting again is that by the end of the process, you will fully understand the code and how it works. This will give you a depth of knowledge that will be hard to develop while using someone else’s code. Perhaps ironically, the process of writing your own version will sometimes help you understand the old code to the point where you no longer need the new version. A computer science PhD colleague of ours tells us that they prefer to write their own version of algorithms to fully understand them, and then, once they have finished they use the existing libraries.

The main downside of writing your own version is that you are replicating work that has already been done, which may be time consuming and unnecessary. However, you are not starting completely from scratch as you have the existing codebase(s) to give you clues as to how to go about achieving what you need. You can read through the existing code and documentation to get insights about how to build your own version. And you can use the old code to test your new code and check that it works the same.

Overall, which codebase to use and whether to start again or not can be a difficult choice. When looking through an old or confusing codebase, it is tempting to throw it all out and start again. It is useful to consider Rule 1 and clarify your goals—what do you really need from the code? What would be the advantages, and disadvantages, of using each codebase or starting again? And you don’t need to decide right away, you can always come back to this decision after spending some time getting to grips with other people’s code.

### Rule 3: Read the documentation

The purpose of documentation is to help people understand the code, so it is worth taking a look. We are not suggesting that you read through all the documentation like a novel, instead use it as a reference. When first starting working with someone else’s code, it can be useful to give the documentation a quick overview to see what is there. And when stuck on a problem, it is worth referring to the documentation to see if your problem is covered. Any particularly important or confusing parts of the code are more likely to be covered. Checking the documentation is useful even if it is wrong or sparse; if there is very little documentation, then, this is an indication that the code probably wasn’t written according to good practices and may not be very reliable.

Documentation includes obvious things like README files or online APIs. And it also includes code comments, docstrings, variable and function names, and any tests that are written. Often good projects will have online documentation that includes tutorials such as a “getting started” section, which is a great place to start.

In the case where documentation does not exist or is incorrect, it can be useful to add your own documentation. This can be very helpful not only for yourself, but also potentially other people working with the code in the future. This does not have to be perfect or too formal. Documentation is essentially just a set of notes about the code, which you will probably find useful to make anyway as you are exploring the codebase. Saving these notes as documentation means that it will be easier to come back to the code after weeks or months and refresh your memory and jump right back in.

Adding to the documentation can be as simple as creating or adding to a README file, which typically explains the functionality at a high level, but may also include installation steps or highlight how to call specific functions via the API. In addition, you could add or change comments within the code itself that explain how specific parts (functions, classes, or individual lines of code) work (or should work). A good rule of thumb is to imagine yourself coming back to the code in a year’s time having forgotten everything and add documentation to help your future self.

### Rule 4: Work out how to run it

A foundational issue when working with other people’s code is to get it to run in the first place. This may seem trivial but in fact can be a very difficult step. Software runs in an environment that includes the operating system, programming language, dependencies, and even hardware. Code that works on one computer might not work on another. This is especially a problem with older code—a recent Nature article challenged researchers with the question of whether they could run research code from 10 years hence, finding issues related to obsolete environments coupled with incomplete documentation [7].

Often the main issue with running other people’s code is using the correct version of the programming language and the code dependencies. Programming languages and individual packages are often updated to add new features and functionality. Sometimes these changes are made with backwards compatibility in mind, so that older code should still work in the new version, but other times this is not the case. Beyond programming languages and dependencies, there may be issues with using a specific operating system, having a specific database installed, or hardware such as graphics cards, sensors, or even requiring a floppy disk drive.

In order to work out how to run the code, a good first step is to simply try and run it as it is in your current environment. If it doesn’t work, then the computer will likely raise an error message that should give you a clue about what is going wrong. You can also look in the documentation. Good documentation will clearly state the intended environment including the programming language and package versions required. Unfortunately, this is not always included. Another option is to use internet searches to work out what versions of the software were the latest releases when the code was written and work backwards from there.

As you work out the specific environment that the code needs to run, you can start replicating that environment on your computer. This might be as simple as using an older version of a programming language and installing specific versions of dependencies. If you do not want to change the environment on your computer, then you can instead create a sandbox to run the code in. A virtual environment allows you to run code with specific versions of a programming language and packages. Beyond that, a virtual machine can emulate an entire computer system (e.g., running a virtual Windows instance on a Mac). There are many options for virtualisation, and the best option will depend on your current computer and the system that you want to virtualise.

It may be that someone else has already worked out a virtualisation setup to run the code. For example, Conda has templates for virtual environments for programming language and package versions that are designed to run specific code. And DockerHub includes Docker templates to set up entire virtual machines. Conda and DockerHub are both community-driven platforms and you can also contribute your own virtualisation setups to help others (and your future self). Even with all this, it might actually be impossible to run the code on your machine. For example, code written to run on a high performance computer such as a supercomputer might simply not be compatible with your laptop.

It may be that you run into issues that are not simply about finding the right environment. For example, it might not be clear what kind of input is required. And some functionality may work okay while other parts of the code raise errors. To confound these issues, the error messages themselves may not give good clues to the problem. In these cases, you will probably need to dig more deeply into parts of the code and try and work out exactly how it works. This can be time consuming and requires patience and persistence. Debugging tools can be a help here, which allow you to track the execution of code step by step and see what kind of resources and data are being used. Of course, there is always the possibility that parts of the code are simply broken, and you may need to do more rigorous testing (Rule 5: Test it does what you expect) or make changes (Rule 8: Think before making changes).

### Rule 5: Test it does what you expect

In 2006, Geoffrey Chang, a rising academic star, retracted several high profile papers in Science due to a software bug [10]. In these papers, Chang had used some code to compute the structures of proteins. Unfortunately this code, which was inherited from another lab, included a small bug that meant that the resulting protein structures were not correct. This problem might have been caught earlier with better software testing. Everyone agreed that the error was unintentional, and fortunately, Chang went on to have a successful academic career, but nevertheless this should serve as a cautionary tale.

When working with someone else’s code, testing is a great way to check what the code does (and doesn’t do) and verify that it actually works. Often a project will already have tests, and finding and running these should be the starting point. These are usually found in a folder simply called “tests” or similar and ideally the documentation will include instructions for running those. Running the tests will further familiarise you with the codebase, and the existence (or lack of) tests also gives you a way to gauge the quality of someone else’s code. You may even find tests that fail—in which case, you could consider making changes to fix the code (Rule 8: Think before making changes).

Beyond the existing tests, you might want to write your own. A good place to start is with a sanity check, a simple example where you can verify that the code gives you the correct answer. For example, one colleague was working with a function to extract words and counts from large pieces of text. They could check this worked in a simple case by passing in the sentence “the quick brown fox jumps over the lazy dog.”

While sanity checks are worthwhile, automated tests are more thorough. This can be as simple as automating a sanity check by writing a test to check that the output of a function is correct. Commonly, this is achieved by using an “assert” statement that raises an error if the output is not what is expected. This is a form of **unit test**, which checks that a specific unit of the software, in this case a function, works as expected. Most programming languages include unit testing functionality or libraries, which include assert statements as well as ways to run many tests quickly and easily.

Another common type of test is a **functional test**—in this case, you can check that the software as a whole does what you expect. For example, you may aim to replicate a figure from a paper, which will act as a kind of test on the software used to generate the analysis for the figure. Functional tests can be a good way to check that the code does what it should do without having to know too much about how the code works.

With both unit and functional tests, you will need some data to check the results with. The codebase may already include some demo data, which is a good first place to start, and also serves as a good example of the data format that the code requires. You may want to add your own testing data, which could be real data, e.g., in the case of replicating a figure. Or you could simulate data, e.g., you could simulate data with known parameters in order to check that a fitting algorithm gives reasonable results. With real or simulated data sets you may not know the “correct” answers and so it is common to use another existing tool to verify the results, where possible.

Overall, the depth and type of testing that you need will depend on your specific goals (see Rule 1: Clarify your goals). In many cases, a few simple sanity checks will be enough to verify that the code is working as expected. More rigorous testing is needed when there are question marks over the quality of the code and when the correct performance of the code is critical.

### Rule 6: Break it down and sketch it out

In software engineering, it is often a good strategy to decompose a problem down into smaller units or modules, and then, put those units together to solve a complex problem. The same thing applies to working with other people’s code. Breaking the code down and working out what each part of it does is often much easier than trying to do it all at once.

**Fig 2:**
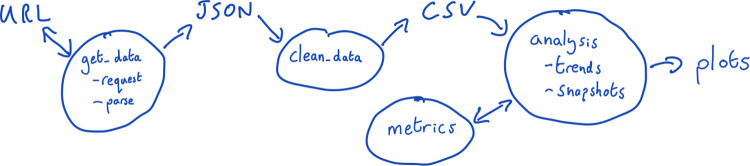
An example rough sketch for a text analysis project. The project involved programmatically downloading data, cleaning that data, analysing it using specific metrics, and producing plots. By sketching it out, you are able to see the general workflow and how different parts of the code can be thought of independently.

A good approach to this method is to draw out how the code works and how the parts interact [8]. This doesn’t need to be perfect and can simply be a quick sketch with a pen and paper or a blackboard (see Fig 2 for an example). You may want to visualise how the code is structured, how data is passed around, which functions call other functions, etc. Just getting something down on paper can help to visualise the system.

Depending on the project and your aims, you may decide to formalise these drawings by designing a figure using visualisation software. You could include this in a write up of a project or add it to the documentation for the codebase. For those interested, you can even use the Unified Modelling Language [11], a generalised way of visualising software systems.

### Rule 7: Ask for help

Research is a collaborative endeavour and it can be easy to forget that help is available—you don’t have to do this on your own. Collaboration is especially useful when working with code, as people have widely varying levels of knowledge and skills in a variety of programming languages and paradigms. Leveraging existing knowledge is a great tactic to increase your own research efficiency. There are many avenues for getting help:

- Google (or other search engines). Software developers rely on search engines to help them code, with 1 study finding that developers spent around 20% of their time searching the web [12]. When it comes to working with other people’s code, Google searches including the name of the project can reveal online resources such as code repositories, tutorials, forums, and more. More generally, Google is a great resource for finding code snippets, short explanations, and diagnosing errors—a good search strategy is to copy and paste error messages directly into Google.
- StackOverflow is a useful resource and is often found in Google search results. This online portal allows users to ask and answer specific coding questions. Almost all of our colleagues use StackOverflow to search for problems they have with code—often someone has already asked and answered a question that you have. If you cannot find a solution through searching, it is also worthwhile asking your own question, and you will often get answers immediately. It might be that the codebase you are working on is too obscure to reference directly. Even then, you can post blocks of code or ask more general questions and get help. Other useful portals include W3Schools and Quora, and there are forums that specialise in specific research fields such as BioStars for bioinformatics.
- GitHub. Many projects will have code hosted on GitHub (see Rule 9: Use version control), which is the industry and academic standard for hosting and sharing code repositories. If you have an issue with the code such as a bug, a suggested new feature, or just a question, then you can raise this on the repository using GitHub Issues, which can then be answered or fixed by the project developers. This is best practice and the preferred way of raising issues for many developers. Before raising an issue, it is worth checking through past issues to see if your problem has been addressed.
- Other project portals. Ongoing software projects often have various online resources to get help. This could include chat platforms such as Slack, Gitter, Discord, and forums. These may be linked from the GitHub page or you can find them by Googling.
- Collaborators. If you have collaborators then ask them. They may be willing to help or may even have used the code before in the past.
- Other researchers. There is a good chance that other people have also worked with the code before. You can check papers that cited the software or associated research articles. If the code is hosted on GitHub, you can check the issues on the repository page or see if it has been forked (see Rule 9: Use version control). Or you can search GitHub for the repository name and look for other versions. If you get lucky, someone might have already done the work of getting to grips with the codebase. They might be happy to send you resources or even come on board as a collaborator.
- The original authors. There is one group of people who, at one point, fully understood the code (hopefully). It is worth getting in touch with the original authors. They might be willing to answer specific questions, they might have some documentation that they haven’t published online, or they might want to collaborate. They will probably be happy that you are using their code. If the code is an ongoing project, then they may even be willing to add functionality that you request or fix a bug.
- Research software engineers. Depending on your institutional affiliations, you may have access to help from a team of research software engineers. This could be in the form of general advice or support or they might be willing to take on a project related to the codebase and work with you.

The act of articulating the problem itself can often lead to the insight that can solve the problem; there are quite a few StackOverflow posts where the accepted answer comes from the original poster! This is sometimes called rubber ducking, named after a programmer who would explain problems to a small yellow rubber duck that they carried with them—just explaining the problem often made the solution more obvious [13].

It may be that you feel so lost that you cannot even articulate the problem. In this case, you can still reach out for help—it is perfectly acceptable to get in touch with someone and simply say that you do not know where to start. If nothing else, they can offer emotional support.

### Rule 8: Think before making changes

When we think about using other people’s code, or software development in general, we think about hacking away and writing code. The temptation is to jump in and start “improving” the code, especially if it is poorly written. But ask if you actually need to make changes. The most efficient way of completing a task is to realise that you don’t need to do it at all. Often changes are necessary but take a second to think first before jumping in.

Making changes can have unintended consequences, with those consequences becoming more likely the less that you understand what the code is doing. What appears to be badly written code may have been written in that way for some important reason. As soon as you change code, you change the ground truth of what the code does and you can no longer be sure if its behaviour is as was intended or was instead caused by your changes.

The book “Working effectively with Legacy Code” [8] identifies four reasons to change code:

- Fixing a bug. Fixing something that is broken.
- Adding a feature. Extending the functionality.
- Refactoring. Changing the design without changing the functionality.
- Optimising. Changing the performance without changing the functionality.

When making a change, the aim is not to break things and to make the change efficiently. For this reason, it is useful to specify exactly what the aim of the change is, and do only that. For example, while fixing a bug you might notice a loop that could be easily optimised—resist the temptation! The efficiency of this loop is probably not important, and every change that you make could cause a serious problem. As renowned computer scientist Donald Knuth says, “We should forget about small efficiencies, say about 97% of the time: premature optimization is the root of all evil” [14].

The best practice when making changes is to write some unit tests before you change anything that cover the existing functionality, as well as tests that will cover any new functionality that you want to add (see Rule 5: Test it does what you expect). Then, once you make the changes you can run those tests to check that you didn’t break anything and that your changes have worked. This can be relatively simple. For example, if you are refactoring a specific function then you can write some automated tests beforehand based on the current outputs of the function (this is called a characterisation test). After you make the changes, you can then run the tests to make sure that the refactoring has not changed the functionality. It is also helpful to describe any changes that you make in the documentation, including what was changed and why (see Rule 3: Read the documentation and Rule 9: Use version control).

### Rule 9: Use version control

Version control is by far the best way to backup, share, collaborate, and track changes with code. In spite of this, we know plenty of colleagues who email each other code, use a shared Dropbox or Google Drive, or do not backup their code at all. If you do not regularly use version control, then this is the most important rule for you.

When we say version control, we usually think of Git and GitHub. GitHub has free accounts that include unlimited code repositories that can be private or public. Other options are available such as BitBucket. If code (or data) is sensitive and cannot be sent to GitHub’s servers, then there are options such as GitLab that allow you to run your own private Git server.

Learning to use Git can feel intimidating but it is actually very simple to get started. You do not need to be an expert and just being able to push and pull changes is all you need to be effective. We will not reproduce here a guide to using Git as there are a wealth of existing resources. For beginners, we recommend working through the PLOS article, “A Quick Introduction to Version Control with Git and GitHub” [15], which should take about an hour. For those more interested, the Pro Git book [16] is a useful resource, alongside many other online guides and tutorials.

You may find yourself in a situation where the code you have inherited is not already stored in a version controlled repository. You could start by moving the existing code into a Git repository and create a starting commit which adds all of the files. Any changes that you yourself make can then be tracked in the usual way with subsequent commits. Doing this ensures it will always be possible to move the code back to a previous state, even when mistakes are introduced, leaving you free to modify the code without fear of losing the original version of the code you inherited.

For advanced users, Git also gives access to a range of useful tools such as GitHub Actions, which allows you to automate parts of your workflow. This can include automated testing to check that code changes have not broken anything and that the tests in the project still pass. Git also allows for easy versioning of code, so that you can rewind a codebase to a specific version or time, which is important for research reproducibility.

### Rule 10: Publish

Depending on your goals, you may have made changes to the code and/or documentation. Why not share those changes? It is often the case that other people will have the same goals from using the code as you had, and your changes could save them a lot of time and difficulty.

The standard for sharing code in both research and industry is GitHub (see Rule 9: Use version control). If the code is already published as an existing GitHub repository, then you can “fork” it to create a copy of the repository with your changes. Depending on what those changes are, you may even make a “pull request” to the existing repository to incorporate your changes in the original codebase. If there is no existing repository, then you can always create a new one.

A small note of caution: Before publishing changes to someone else’s code, it is a good idea to check that this is allowed in the software licence. The licence will often be in the main direct project directory as “LICENSE.txt” or similar. While much of research software is published under permissive open source licences, this is not always the case and should be checked. And even open source licenses may have conditions such as attribution to the original author.

Beyond selflessly contributing to the research community, sharing changes can help your own career. Publishing code online will build your online profile and reputation, which can be helpful when applying for jobs or other opportunities. Other researchers who see your code may reach out to collaborate. And you may even have other people contribute to and improve your codebase, or even just identify bugs that you missed.

Publishing code is a good practice to get into. Often if we know that code will be published, then we write it to a higher standard, and avoid lazy shortcuts that can cause problems down the line for ourselves and others. There is an increasing movement in research to publish code alongside research projects [6], and this is much easier if you are already in the habit. Publishing code will also make it easier for others, and yourself, to replicate your work at a future date. One of our colleagues tells a story of being contacted several years after a paper was published to collaborate on a similar project, a great opportunity which they almost had to turn down—they couldn’t find their code because it was written on an old laptop that they no longer had access to. Luckily, they realised that they had published the code on GitHub and as such the collaboration was possible.

## Conclusion

Reflecting on working with other people’s code can help you to write better code too. Being exposed to different coding practices, design patterns, tools, etc., will broaden your programming knowledge. And importantly, negative experiences and frustrations can act as visceral lessons in what not to do.

Working with other people’s code is an important part of modern academic life in many fields and will continue to be so. It can be a frustrating, difficult, and challenging experience. It is worth spending some time thinking about how best to approach this common problem. We hope that this article has served this purpose and that the reader has taken away at least some small tip about working with other people’s code.
